# Plasma galectin-9 levels correlate with blood monocyte turnover and predict simian/human immunodeficiency virus disease progression

**DOI:** 10.1186/s41231-023-00160-w

**Published:** 2024-01-02

**Authors:** Laurent Zablocki-Thomas, Amir Ardeshir, Naofumi Takahashi, Kevin S. White, Cinar Efe Sumer, Zoey K. Wallis, Elizabeth S. Didier, Woong-Ki Kim, Kenneth C. Williams, Marcelo J. Kuroda

**Affiliations:** 1 Department of Anatomy, Physiology, & Cell Biology, School of Veterinary Medicine, and California National Primate Research Center, University of California, Davis, County Road 98 & Hutchison Drive, Davis, CA, USA; 2 Division of Microbiology, Tulane National Primate Research Center, Covington, LA, USA; 3 Department of Microbiology & Immunology, Tulane University School of Medicine, New Orleans, LA, USA; 4 Joint Research Center for Human Retrovirus Infection, Kumamoto University, Kumamoto, Japan; 5 Biology Department, Boston College, Higgins Hall 468, 140 Commonwealth Avenue, Chestnut Hill, MA, USA

**Keywords:** Simian immunodeficiency virus, Galectin-9, Macrophage turnover, Rhesus macaque, Pathogenesis

## Abstract

**Background:**

Late-stage human immunodeficiency virus (HIV) infection is typically characterized by low CD4 + T-cell count. We previously showed that profound changes in the monocyte turnover (MTO) rate in rhesus macaques infected by the simian immunodeficiency virus (SIV) outperforms declining CD4 + T-cell counts in predicting rapid health decline associated with progression to terminal disease. High MTO is associated with increased tissue macrophage death. However, MTO analysis is complex and not directly applicable to humans.

**Methods:**

We explored blood-available biomarkers associated with MTO using comprehensive immune cell profiling via flow cytometry, blood cell count and chemistry, and ELISA.

**Results:**

Plasma galectin-9 was identified as the most highly correlated marker with MTO. This correlation remained statistically significant during acute, chronic, and late-stage infections caused by three different SIV strains tested. In addition, the galectin-9 level also predicted decline in animal health, requiring medical cull. The correlation between MTO and galectin-9 was maintained even in uninfected animals showing variable MTO.

**Conclusions:**

These findings support the exploration of galectin-9 as a surrogate biomarker of MTO for non-invasive monitoring of disease progression (e.g. HIV) that may also be applicable in humans and as a potential indicator of tissue macrophages apoptosis.

## Background

Declining CD4 + T-cell count is a key factor for AIDS diagnosis and helps to determine the need for associated prophylaxis measures [1]. Our lab previously demonstrated that a rapid CD4 + T-cell decline predicted progression to AIDS less reliably than increasing monocyte turnover (MTO) [2, 3]. Using SIV-infected rhesus macaques as a model for HIV infection, monocyte turnover was monitored by incorporation of the thymidine analogue, 5-bromo-2’-deoxyuridine (BrdU) into dividing cells. MTO was then defined as the percentage of BrdU-labeled cells among CD14 + blood monocytes, 24 h post BrdU pulse. As previously reported, increasing MTO reflected increased monocyte production as well as higher rate of apoptosis and arrival of new macrophages in lymph nodes and lungs [3, 4]. Furthermore, in lungs of SIV-infected macaques, increased macrophage infection and lesion severity correlated positively with MTO [4, 5]. In the jejunum and colon, high MTO was associated with a loss of CD206^+^ macrophages [6]. Thus, using BrdU incorporation to identify dividing cells at the time of pulse has been useful for monitoring cell trafficking as well as disease progression in SIV-infected macaques.

There exist limitations to applying thymidine analogue staining methods in vivo, especially in humans. These include administration, repeated blood collections required for data acquisition, toxicity, and limited publications regarding monocyte production and turnover rates. Only one other model, to our knowledge, reported the observation of early BrdU-labeled monocytes in blood whereby Ye Chean et al. showed an apparition of a BrdU+ population of monocytes 8 h after pulse in the context of bacterial peritonitis in mice [7]. These BrdU+ monocytes were pre-monocytes released from the bone marrow that retained their proliferation capacity in the blood. In SIV, MTO was associated with tissue macrophage death [4].

Although MTO appears to be an outstanding biomarker for tissue macrophage loss and replacement, it is not readily applicable for use in humans mainly due to potential toxicity. Since macrophages play central roles in tissue homeostasis and inflammation, a non-invasive method that relates to monocyte turnover would potentially benefit human health applications. Toward this goal, we identified galectin-9 as a possible surrogate. Galectin-9 is a tandem-repeat lectin composed of two carbohydrate-binding domains connected by a linker peptide [8]. Galectin-9 is present in the nucleus, cytoplasm, cell surface and extracellularly. It binds to many different surface proteins as well as extracellular matrix. This lectin also can be secreted from cells and may be detected in the plasma. One of the most studied functions of galectin-9 is its capacity in vitro to bind to Tim-3 at the surface of Th1 cells and induce apoptosis [9].

Plasma galectin-9 has gained particular interest over the past decade for its use as a biomarker in association with viral infections such as HIV, dengue, hepatitis B & C, influenza, human cytomegalovirus, chikungunya fever, coronavirus, as well as parasitic and bacterial infections such as malaria, leptospirosis, and tuberculosis [10, 11]. In most of these cases, plasma galectin-9 levels reflected not only the presence of infection but also its severity. Interestingly, elevated galectin-9 levels also were associated with autoimmune disorders, cancer, and other conditions [12]. In relation to HIV infection, plasma galectin-9 levels also appeared to predict onset of AIDS and death within 12 weeks in HIV-infected patients [13]. Furthermore, plasma galectin-9 levels correlated with viremia in HIV patients receiving antiretroviral therapy (ART) [14], as well as with viral load and CD4 + T-cell count [15]. In ART-treated patients, plasma galectin 9 was a predictor of adverse non-AIDS events [16].

To determine if galectin-9 may serve as a surrogate biomarker of MTO that reflects disease progression, we aggregated data from SIV-infected rhesus macaques that were studied in our previous publication [2]. Identification of such markers would help monitor the tissue macrophage dynamics and turnover during the course of HIV/SIV infection. In addition, such a marker could help overcome the challenges inherent to MTO measurement and cell turnover identification, which is currently not feasible outside the animal model.

## Methods

### Animals

All animals usedin this study were born and housed at Tulane or California National Primate Research Centers (TNPRC or CNPRC) as previously described [2]. Animal details are provided in Supplementary Table S1. All aspects of the management and experimental use of animals complied with USA federal regulations and guidelines published in the US National Research Council *Guide for the Care and Use of Laboratory Animals (2011)* [17], and the US Department of Agriculture Animal Welfare Act and Animal Welfare Regulations [18].

### Medical cull

Humane euthanasia decisions were made exclusively by the veterinary care team, taking in account only the current animal status. The authors and the research team had no role in this decision making. Euthanasia protocols were performed using the approved method described by the AVMA guidelines for euthanasia of animals [19].

### Animal inoculations and treatments

Animal inoculations and treatments were described previously [2]. Briefly, animals were inoculated intravenously with SIVmac251, SIV0302-2, or SHIV89.6P. Some animals received anti-CD8 antibody (Supplementary Table S1) subcutaneously (10 mg/kg) on day 6 post infection and intravenously (5 mg/kg) on days 8 and 12. Some animals also received ART drugs, PMPA (20 mg/kg per day) and FTC (50 mg/kg per day) (Supplementary Table S1). Animals treated with liposomes received two doses three days apart. The first dose was by aerosol exposure using 10 ml of 3.3 mg/ml Clophosome^®^_,_ a neutral liposomal clodronate (FormuMax, cat. F70101C-N). The second dose was injected intravenously (I.V.) using 8 mg/kg of Alenphosome^™^, alendronate liposomes (FormuMax, cat. F70103A-N). BrdU injections for pulse chase labelling of dividing cells was performed immediately prior to I.V. liposome injections. MTO and galectin-9 levels were measured one day post I.V. liposome injection.

### Biomarker measurements

Plasma viral load, MTO, immunostaining, and flow cytometry procedures were as described previously [2]. In brief: SIVmac251 plasma viral loads were quantified using branched-DNA amplification, and other strains of SIV and SHIV were quantified using RT-qPCR, targeting SIVmac239 gag sequence. For MTO measurement: the thymidine analog, BrdU (Sigma-Aldrich, St. Louis, Missouri, USA) was injected I.V. at 60 mg/kg per animal and the percentage of BrdU-incorporated monocytes and macrophages were counted in blood 24 h later [2]. For the immunostaining and flow cytometry procedures, briefly, 200 μl of EDTA–anticoagulated whole blood was washed with PBS and incubated with surface monoclonal antibodies at room temperature for 20 min (Table S2). Red blood cells (RBCs) were lysed with FACS-lysing solution (BD Bioscience, San Jose, California, USA). To analyze BrdU incorporation, cells were permeabilized using Cytofix/Cytoperm per manufacturer’s instructions (BD Biosciences), incubated with DNase I (Sigma-Aldrich) at 37°C for 1 h and then stained with anti-BrdU antibody for 20 min at room temperature. After washing, cells were fixed in 250 μl of 1% paraformaldehyde in PBS. Stained samples were acquired using LSRII, LSRFortessa, FAC-SAria or FACSymphony (BD Biosciences). Acquired data were analyzed using FlowJo software (FLOWJO, LLC) [2]. To measure plasma factors, EDTA anti-coagulated blood was centrifuged at 2,100 × g to recover the supernatant that was then centrifuged a second time at 2,100 × g followed by storage of plasma in aliquots at −80°C. ELI-SAs for sCD163 (IQ Products, IQP-383, plasma diluted 1:100), galectin-3 (R&D Systems, DGAL30, plasma diluted 1:10) and galectin-9 (R&D Systems, DGAL90, plasma diluted 1:10) were performed on frozen plasma following manufacturer’s recommendations.

### Statistical analysis

All analyses were performed with R version 4.2.2. Statistical tests were performed as described in the figure legends. *P* values were not corrected for multiple tests unless specified. A *P* value less than 0.05 was considered significant.

## Results

### Few markers correlate with MTO increase in end-stage SIV-infected animals

**Fig. 1:**
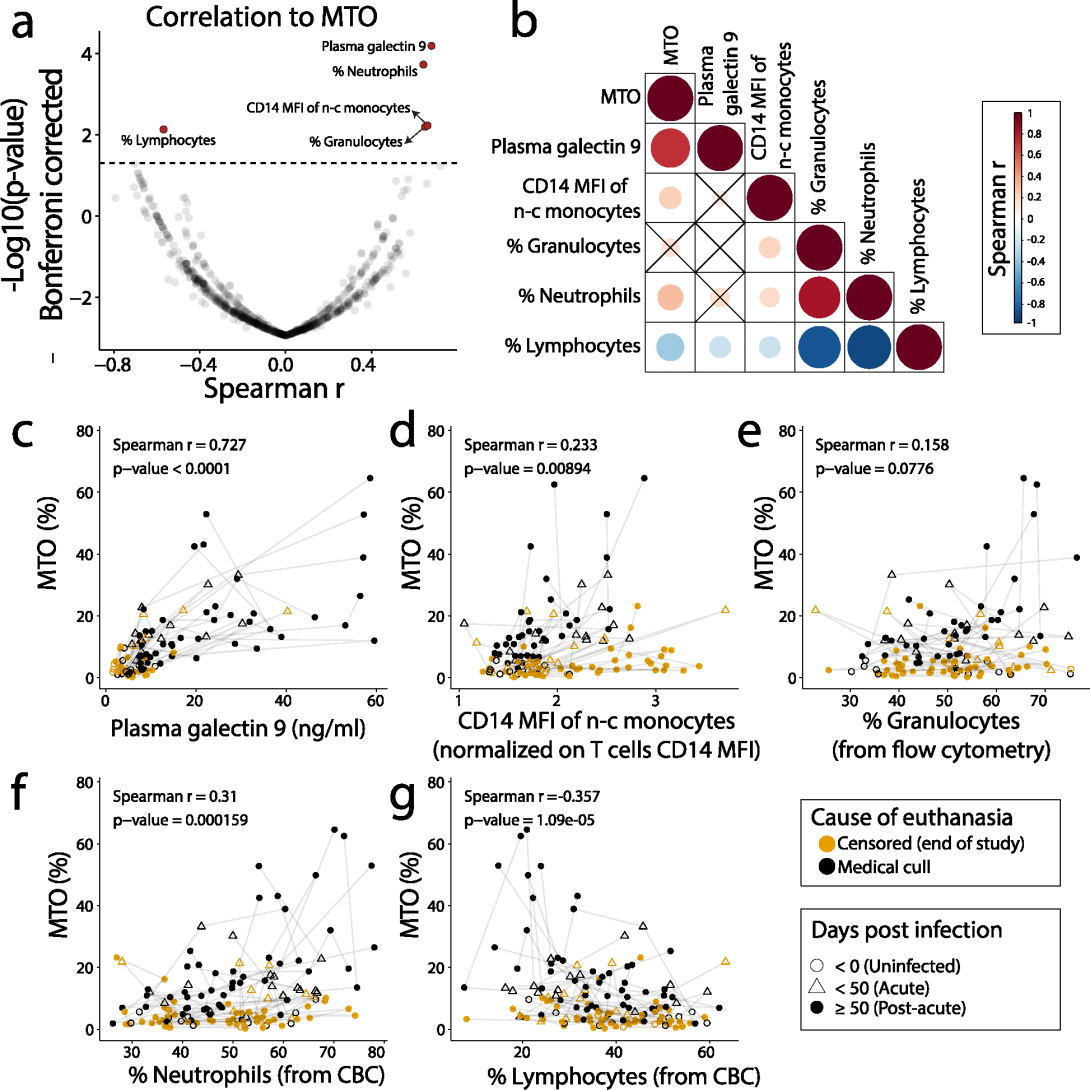
Plasma galectin-9 correlates strongly with MTO during SIV infection. **a** Spearman r and Bonferroni corrected *p*-values of MTO correlation with 865 different markers were measured from longitudinal samples collected from 22 SIV-infected animals. The horizontal dashed line indicates the adjusted *p* value of 0.05. The calculation only included time points of the post-acute phase (≥ 50 days post-infection) from animals ultimately requiring medical cull. **b** Correlation matrix for MTO and the five BrdU-independent markers identified in panel **a**. Circle size and colour represent correlation strength and direction, respectively. Circles were drawn only for *p* value < 0.05. Contrary to panel **a**, the calculation included all available time points from all animals. **c-g** Measured MTO rates in relation to the five identified markers. Lines connect time points for each animal in chronological order. All available time points are represented. Spearman r and *p* values are the same as calculated for panel **b** for all animals and time points. Plain black circles are the time points used for panel **a**

We previously determined that increased MTO outperformed low CD4 + T cell counts and high viral load for predicting terminal disease progression requiring medical cull in SIV-infected rhesus macaques [2]. Since measuring MTO requires specialized expertise and is difficult to apply to humans, we explored possible mechanisms of MTO increase that could be applied to identify surrogate biomarkers for evaluation. To address this, we measured multiple parameters on specimens collected longitudinally from the rhesus macaques infected with SIV (Supplementary Tables S1 and S3). To limit the impact of missing values at each MTO time point, the average value of each parameter was calculated within a 15-day window. All of these markers, with the exception of body weight, were derived from the blood specimens, including complete blood cell count (CBC), blood chemistry, flow cytometry, plasma viral load, galectin-3, -9, and soluble CD163 (sCD163). Some of the parameters used in comparison to MTO were co-linear or as a combination of markers. Since MTO increased in animals ultimately requiring medical cull, we selected time points during the post-acute phase of these animals. This would ensure that the biomarkers of interest would be associated with MTO increase and not the steady rate MTO occurring during non-progressing SIV infection or in uninfected animals. Of the 865 tested markers, including cytokines and chemokines and others associated with HIV comorbidities and myelod cells like gal-3, IL-18 and sCD163, only five significantly correlated with MTO after Bonferroni correction of the *p* values. These markers were plasma galectin-9, CD14 mean fluorescence intensity (MFI) of non-classical (n–c) monocytes (normalized from T cells CD14 MFI), % granulocytes determined by flow cytometry, % neutrophils and % lymphocytes determined by CBC (Fig. 1a and Supplementary Fig. S1b–f). The last three indicators, % granulocytes, % neutrophils, and % lymphocytes, also correlated to each other (Supplementary Fig. S1a). Interestingly, typical markers of disease progression such as % central memory (CM) CD4 + T cells, plasma viral load, ratio of CD4 +/CD8 + T cells, sCD163, and galectin-3 failed to significantly correlate with MTO once corrected for *p*-value (Fig. 1a, Supplementary Fig. S1g–j).

### Galectin-9 best correlated with MTO

We next extended this analysis by repeating the correlation test at each available time point to include data from specimens of uninfected animals, from animals during the acute phase of SIV infection, and from animals requiring euthanasia. At most of these time points, MTO remained at basal levels (Supplementary Fig. S2). Among the significant non-BrdU-dependent markers identified in Fig. 1a, galectin-9 had the strongest correlation with MTO (Fig. 1b–g). Here again, the percentages of granulocytes, neutrophils and lymphocytes highly correlated to each other. We also noted that some animals exhibited increased galectin-9 before increased MTO (ER17 and FI38) and others displayed increased MTO prior to increased galectin-9 (GK40 and IT27) (Supplementary Fig. S1b). Thus, it remains unclear which factor increases first or influences the other factor to increase.

### Galectin-9 correlated with MTO regardless of disease phase or viral strain

**Fig. 2:**
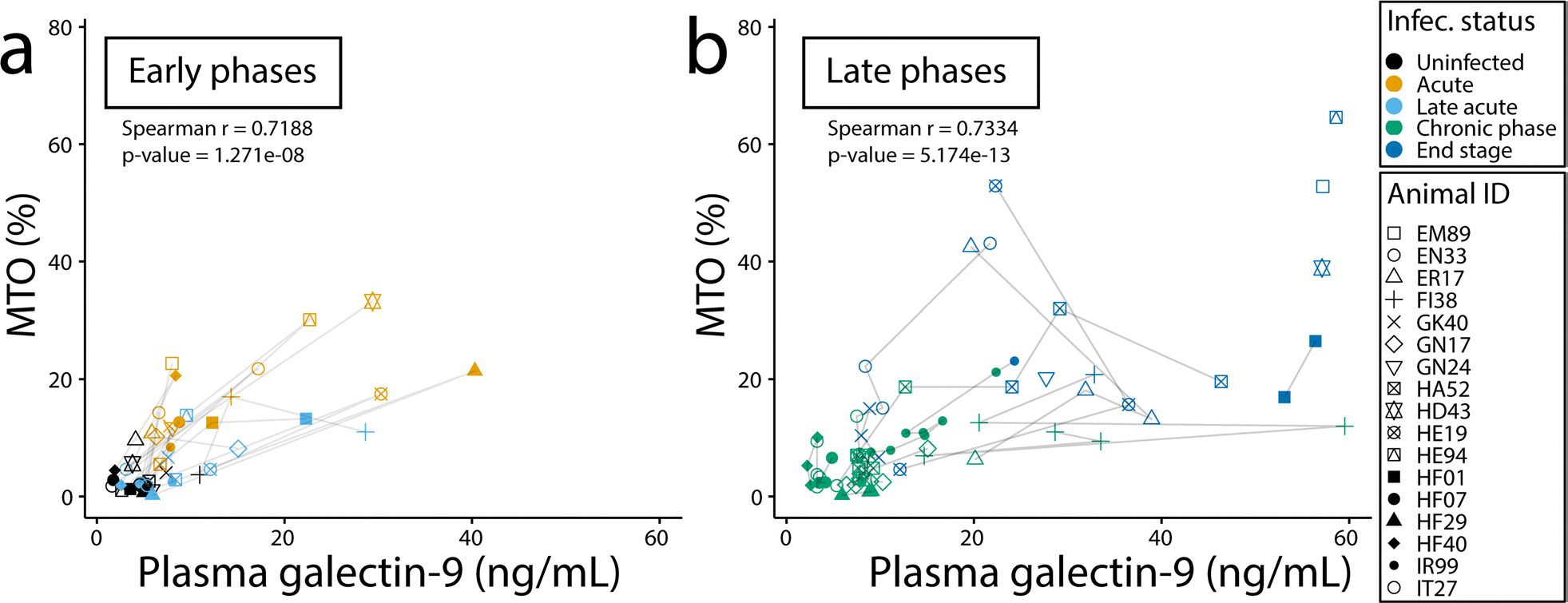
Plasma galectin-9 correlates with MTO during the acute and late phases of SIV infection. Correlation of galectin-9 with MTO during early (**a**) and late (**b**) phase of SIV infection. Time points were classified based on the time post-infection and cause of euthanasia (medical cull or end of study). Uninfected values were from animals ranging from 54 to 40 days before infection. Acute phases are from samples at 9 days post-infection (DPI), except for EN33 and HE94, which were at 13 DPI. Late acute represents the first time point after the acute phase (ranging from 49 to 62 days post-infection). Chronic phase time points were higher than 50 DPI and at least 100 days prior to a medical cull. The end-stage marks the time points less than 100 days prior to a medical cull. Data points between 50 and 63 DPI are present in both Panels **a** and **b** as late acute and chronic stage, respectively

Frequent measurement of MTO is not feasible since it requires a complete blood cell turnover of CD14 + monocytes in order to “reset” BrdU cell incorporation for accurately measuring only the newly-produced CD14 + monocytes, a process requiring a 14-day washout period [20]. Thus, while there was limited data on increased MTO during the acute phase of SIV infection, we were still able to test if galectin-9 correlated with MTO during acute and late phase infection. Interestingly both phases correlated with MTO in a similar fashion (Fig. 2). The animals were also infected by three different viral strains (Supplementary Table S1) that produced different dynamics of infection progression. For example, CD4 + T-cell depletion was very rapid and almost absolute in the animals infected with SHIV89.6P compared to observations in animals infected with other strains of SIV [2]. Regardless of these differences, there remained a direct correlation between MTO and galectin-9 during the acute and late phases, with the exception of animals with SIV0302-2 during late stage infection (Supplementary Fig. S3). This exception may reflect the lower number of specimen samples available during high MTO time points thereby limiting the analysis sensitivity.

### Galectin-9 is a non-invasive binary classifier of disease state

**Fig. 3:**
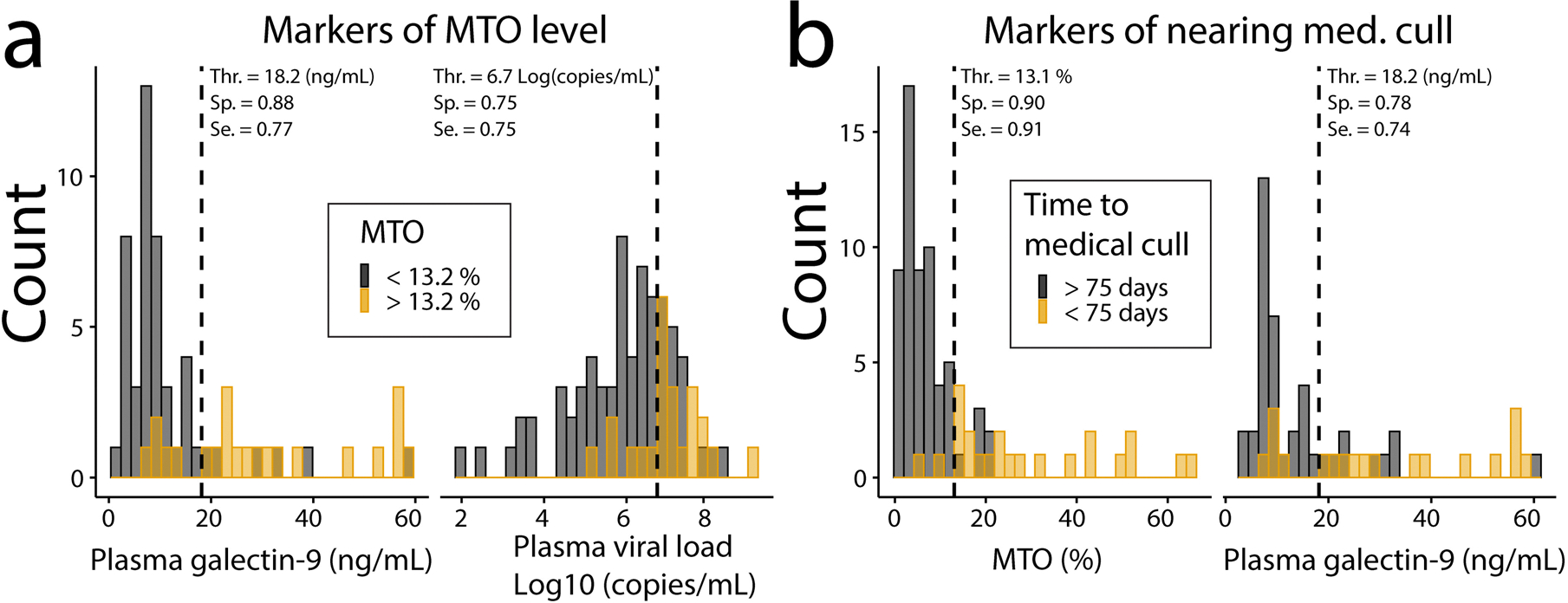
Plasma galectin-9 outperforms viral load in predicting future disease progression. Histogram of post-acute time point (DPI ≥ 50) for SIV-infected animals. Samples were categorized based on MTO level (**a**) or nearing euthanasia (**b**). **a** Among 9 biomarkers tested, the two best markers for classifying samples in higher or lower MTO groups, i.e. MTO higher or lower than 13.2% as estimated previously [2], were plasma galectin-9 and plasma viral load. **b** Among 10 biomarkers, the two best markers to classify samples based on time to medical cull were MTO and plasma galectin-9. ROC curves were calculated, and the optimal threshold was determined with Youden’s J statistic to maximize the sum of sensitivity and specificity. In case of a tie, thresholds with the best specificity were selected. The dashed lines represent this threshold. Thr. = threshold value for the measured parameter, Sp. and Se. = specificity and sensitivity at the threshold, respectively. If time to medical cull could not be determined due to the end of the study censorship, samples were not included in panel **b**

We next focused on the classifier power of each variable listed in Supplementary Fig. S1a at a post-acute SIV infection time point (> 50 days post infection). Plasma galectin-9 levels best correlated with MTO rates (Fig. 3a, Supplementary Table S4). For example, in specimens with plasma galectin-9 levels higher than 18.2 ng/ml, there was an 88% probability that the animal also exhibited an increased (> 13.2%) MTO rate at that time point. The rate of 13.2% MTO was determined previously by machine learning algorithm modelling to predict onset of terminal disease progression in SIV-infected animals [2]. In specimens with plasma galectin-9 levels below the threshold of 18.2 ng/ml, there was a 77% probability that the MTO rate at that time point fell below 13.2%.

We also examined when animals were deemed by veterinarians to require medical cull due to clinical signs (e.g. lethargy, significant weight loss). We found that along with increased MTO rate, plasma galectin-9 level was the second best criterion the correlated with medical cull time point (Fig. 3b, Supplementary Table S5). When MTO was higher than 13.1% at a given time point, there was a 90% probability of that animal requiring medical cull within 75 days. Conversely, when the MTO was lower than 13.1% there was a 91% probability that the animal did not require medical cull within 75 days of that time point. We next tested whether MTO or plasma galectin-9 levels at any time point post-acute phase would correlate with medical cull (Supplementary Fig. S4, Supplementary Table S6). At any time point post-acute phase, an MTO rate higher than 6.6% or plasma galectin-9 level higher than 9.2 ng/mL produced 82% and 90% probabilities, respectively, that the animal would require medical cull. In animals with values below these thresholds, there were 85% or 69% probabilities, respectively that the animal avoided medical cull during the time of the study. It is worth noting that some animals may have required medical cull if the study continued for a longer period of time, which is a limitation of this analysis.

Overall, MTO rate correlated better with days to euthanasia or medical cull than did plasma galectin-9 levels (Supplementary Fig. S1k&l). It appears that MTO was less variable compared to plasma galectin-9 levels. Plasma galectin-9 levels may change more rapidly in response to environmental stimuli. This emphasizes the relevance in MTO and galectin-9 biomarkers for assessing infection outcomes and their progression rates. These markers exhibited the highest area under the receiver operator curve (AUC of ROC) (Supplementary Tables S4–6) among the 10 variables tested. While galectin-9 may not be as predictive as MTO at a given time point, it is less invasive to analyse this marker and can be tested on repetitive specimens for more effective evaluation compared to measuring MTO which is more difficult to assess in humans.

### MTO rate and galectin-9 levels correlated to each other in uninfected animals

**Fig. 4:**
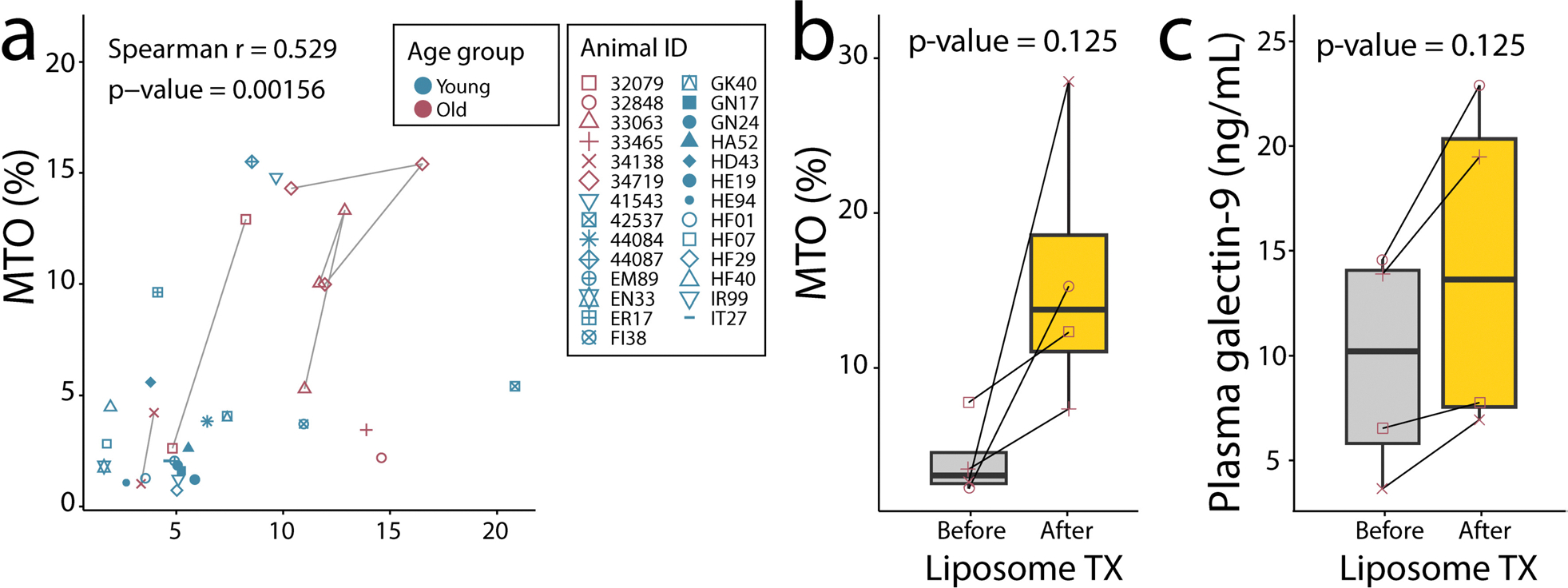
Plasma galectin-9 correlates with MTO in animals not infected with SIV. **a** Spearman correlation of plasma galectin-9 with MTO in uninfected animals. **b** and **c** Changes in MTO and plasma galectin-9 levels, respectively before and after animals received bisphosphonate liposomes via aerosol four days before sample collection, and I.V. one day before sample collection. Before time points represent the average of the available baseline values for each animal. Paired Wilcoxon test *p* values are indicated, but power analysis for significance was not possible with the few available paired samples. The upper and lower whiskers represent the minimum and maximum data points. The length of the box is the difference between the 75th and 25th percentiles, and the line within the box represents the median. TX = treatment

While MTO and plasma galectin-9 levels correlated during the course of SIV infection in rhesus macaques, it remained unclear if this association existed in uninfected animals. We gathered archived samples from animals not infected with SIV experiencing unexplained fluctuating MTO levels. We demonstrated again that from all tested samples, MTO rates and plasma galectin-9 levels significantly correlated to each other (Fig. 4a and Supplementary Fig. S5a and d). Interestingly, there was no significant correlation in each age group of young and old animals, although this may reflect a lack of sufficient numbers samples with high and low levels in the varying age groups of animals (Supplementary Fig. S5b, c, e and f). We also treated animals with liposome-bisphosphonates to deplete monocytes and macrophages to mimic a state of high MTO. Liposome-bisphosphonate treatment was associated with an observable, reproducible increase of MTO and galectin-9 (Fig. 4b and c), however statistical significance is not possible with only four pairs of data using Wilcoxon paired test.

## Discussion

We previously showed that increased MTO rate strongly associated with terminal disease progression in SIV-infected rhesus macaques, regardless of the viral strain used [2]. In the current study using the same set of animals, we analysed longitudinally the level of plasma galectin-9 in relation to MTO, other biomarkers and clinical factors. Similar to previously published studies by others [13, 21], we found that plasma galectin-9 increased during the acute phase and again during the late stage of SIV infection. We demonstrated that galectin-9 plasma levels also directly correlated with MTO rate, regardless of disease stage or viral strain. Galectin-9 and MTO appeared to be more sensitive than plasma viral load or CD4 +/CD8 + T-cell ratio in relation to assessing disease stage, and future clinical outcome. Furthermore, in uninfected animals treated with monocyte/macrophage-depleting liposomes there occurred an associated galectin-9 increase in plasma. Although the number of samples was insufficient for reaching power or statistical significance, this supports the mechanism that destruction or loss of macrophages relates to increases in plasma galectin-9.

The question about a potential direct link between MTO and plasma galectin-9, however, remains unproven. Galectin-9 is considered an alarmin that is secreted in damaged tissue and contributes to inflammation and neutrophils infiltration [22]. Galectin-9 may be an important factor that induces increased monocyte production and migration, although this has not been reported yet. There was no consistent trend regarding the chronology in the increase of each factor in that sometimes MTO increased first followed by increased plasma galectin-9 and vice versa. This raises a question whether one triggers the other or if both biomarkers increase in response to a broader disease process.

Galectin-9 has been recently considered a biomarker of disease severity based on its involvement in host–pathogen interactions, increased plasma levels in infectious and autoimmune disease, and ability to cause cell death [12]. In fact, galectin-9 can trigger monocyte apoptosis in vitro [23], but its effect on monocytes/macrophages in vivo, especially locally in tissues, is less well understood. Interestingly, monocytes themselves produce galectin-9 in vitro and this increases in the presence of Dengue virus infection [24]. The mechanism of galectin-9 increase in vivo is not well understood although many cells are capable of producing galectin-9. Neutrophils shed galectin-9 during HIV and SARS-CoV-2 infections [25, 26]. The trigger of this shedding however, still is unknown, as is its actual contribution to the total plasma galectin-9. It is worth mentioning that monocyte-derived macrophages express even more galectin-9 than monocytes [27], and that cells from the gut epithelium as well as macrophages are the cells that express galectin-9 the most [28, 29]. The observed macrophage apoptosis in late SIV infection could be responsible of the galectin-9 increase. This could be due to leakage from apoptotic bodies which are less likely to be cleared by macrophages due to their destruction. Further studies will determine precisely how SIV and other infection may induce increased production of galectin-9.

The mechanism of MTO increase also remains unclear. From all of our studies, there was a direct link between increased monocyte turnover and destruction of tissue macrophages in relation to terminal disease progression in SIV-infected nonhuman primates [3–7]. An increase in MTO would likely be in response to the increased demand for tissue macrophages. However, MTO increase cannot be a simple boost in monocytes availability since the production capability of the bone marrow is not necessary linked to the speed at which newly-produced monocytes leave the bone marrow (which is what MTO measures). Results reported by Teh et al. (2022) using a mouse model described that tissue disruption resulted in increased macrophage turnover [7]. This led to an LPS-dependent production of premonocytes from the bone marrow. These premonocytes were actually dividing in blood and in tissues for providing a future stock of tissue macrophages to help restore tissue homeostasis. In the case of SIV infection, we know that when MTO is high, tissue macrophages are infected and die. This could lead to tissue disruption, blood LPS increase and premonocyte secretion from the bone marrow. This is supported by the finding that LPS increase has indeed been reported in HIV/SIV infection [30].

MTO and plasma galectin-9 are markers of health deterioration, but what role do they play? If MTO indicates actual stress on the tissue macrophage, then the mechanism in disease pathogenesis relates more directly to tissue macrophage turnover and indirectly on MTO. Premature macrophage death thus probably contributes to the loss of tissue homeostasis as observed in HIV/SIV infection and as monitored by plasma LPS increase [30]. Galectin-9 could act at several stages to further HIV/SIV infection pathogenesis. This factor facilitates differentiation of macrophages into a state that suppresses T-cell proliferation thereby reducing inflammation [31]. Galectin-9 mediates HIV-1 transcription and latency reversal in vitro and ex vivo [32, 33], as well as enhances T-cell migration and HIV-1 infection of T cells via PDI [34]. Galectin-9 also has been shown to increase viral susceptibility of host cells [35]. Thus, on the one side, increase of MTO is most likely a marker of tissue macrophages destruction. On the other side, increased galectin-9, could be a factor in the rapid health decline observed in the end stage of SIV infection. However, MTO and galectin-9 increase are not markers of unstoppable disease progression as they can be reversed as seen in the acute phase.

The putative role of galectin-9 during the course of the SIV/HIV infection is still unclear. One aspect that is rarely taken in account is the chemical status of the galectin-9. The cleaved form is not capable of interacting with or linking two carbohydrate moieties due to the presence of only one carbohydrate-binding domain (CBD). Experiments have shown that cleaved galectin-9 was unable to induce T-cell death or act as an eosinophil chemoattractant [36, 37]. The commercially-available kits measure both cleaved and non-cleaved forms of galectin-9 which would limit interpretation of plasma galectin-9 functionality. Recent measures of specific full-length and cleaved galectin-9 forms showed that AIDS was mainly characterized by a specific increase in the cleaved form of plasma galectin-9 [13], although the full-length form was also significantly increased. More studies will be needed to determine if other forms of galectin-9 are present and active during SIV/HIV infection.

The similar associations between MTO and galectin-9 levels during acute and late phase SIV infection could be due to similar mechanisms. During both stages there occurs relatively uncontrolled SIV infection. In the acute phase, the lack of controlled virus replication would be due to the inherent delay in establishing adaptive immunity. In the late phase, the lack of control could be imputed to the progress of the infection and its destructive effect on the adaptive immunity. The higher SIV/HIV infection levels during these two phases would lead to increased demand in monocyte/macrophages and increased inflammation that in turn, produce tissue damage thereby leading to galectin-9 production and release. Without resolution of infection during late stage SIV infection, MTO and plasma galectin-9 keep increasing until medical cull is required based on declining animal health condition.

## Conclusion

This study highlights a role of galectin-9 as a potential SIV/HIV disease status biomarker. We also demonstrated for the first time that plasma galectin-9 directly associated with MTO that we earlier found to serve as an indicator of macrophage death and turnover in tissue. This supports a potential role for plasma galectin-9 as a surrogate biomarker of tissue damage. While more studies are required to associate galectin-9 with tissue macrophage stress, the results of this study offer the potential for applying plasma galectin-9 measurement as an invaluable tool and alternative to MTO analyses, to estimate the status of tissue macrophages.

## Supplementary Material

Supplementary Figures

## Data Availability

Data and code will be made available freely from the corresponding authors upon reasonable request.

## Abbreviations

MTO: Monocyte turnover
CNPRC: California National Primate Research Center
TNPRC: Tulane National Primate Research Center
ART: Antiretroviral therapy
NHP: Non-human primate
BrdU: Bromodeoxyuridine
MFI: Mean fluorescence intensity
CBD: Carbohydrate-binding domain
