# Supplementary material for: Plasma galectin-9 levels correlate with blood monocyte turnover and predict simian/human immunodeficiency virus disease progression
Source: Transl Med Commun. Author manuscript; Available in PMC 2025 Jan 16. (PMC11737433; doi:10.1186/s41231-023-00160-w)
Supplement: Supplementary Figures [file NIHMS2042644-supplement-Supplementary_Figures.docx]

**Supplementary Figures and Tables**


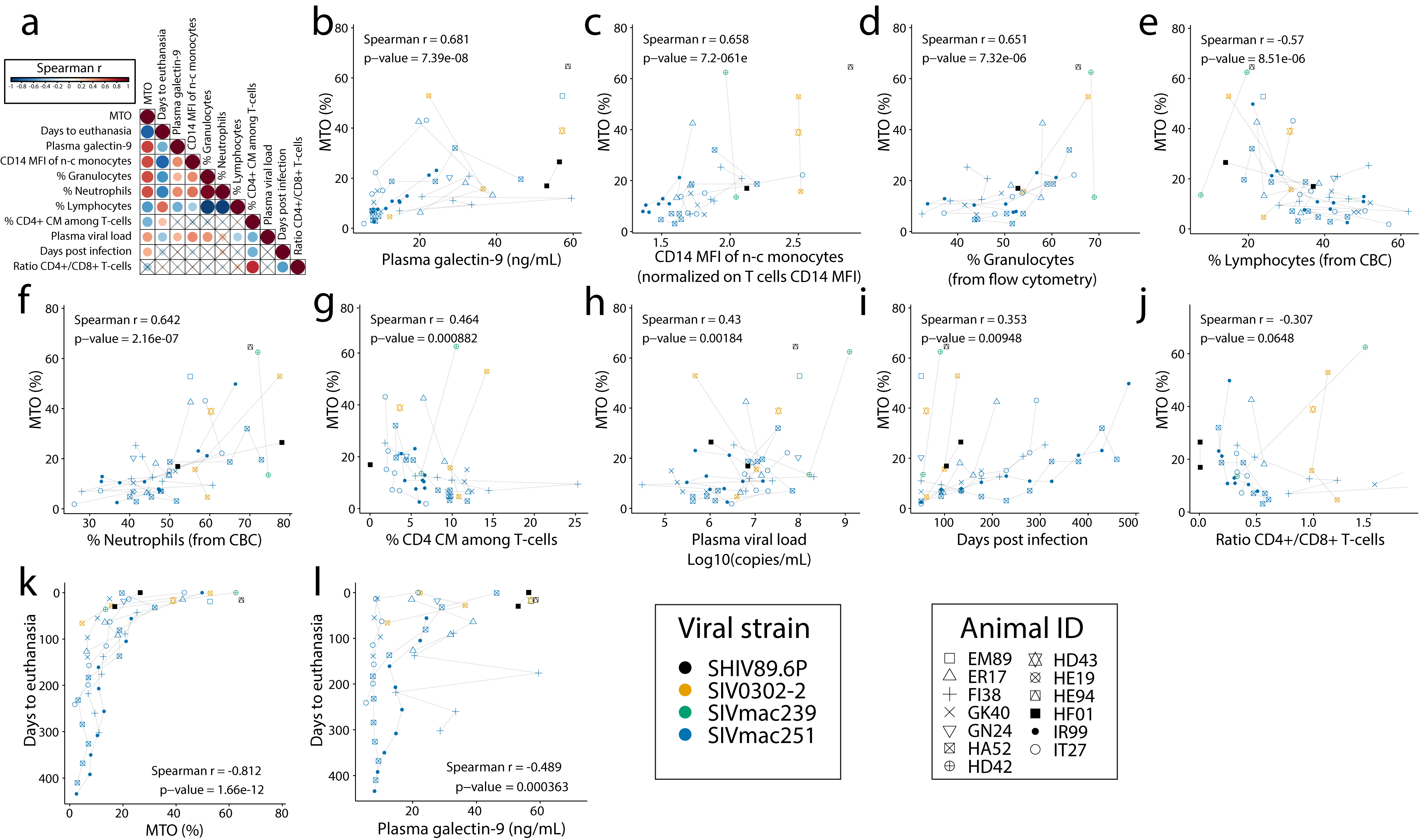


**Figure S1. Galectin-9 correlates with MTO during post-acute phase of SIV infection.** (a). Correlation matrix for MTO and the six BrdU-independent markers identified in Fig. 1 and four classical markers of disease progression. The circle size represents Spearman r. An X indicates non-significant *p* value (p value > 0.05). These time points were the same as applied in Fig. 1a, *p* values are not corrected. (b-j). Evolution of MTO for the markers shown in a. Lines connect time points for each animal in chronological order. Only time points used for a are represented. Spearman r and *p* value were the same as calculated for a. (k-l) Correlation of MTO and plasma galectin-9 with days to euthanasia. a-l Calculation only included time points during the post-acute phase (≥ 50 DPI) from animals ultimately requiring euthanasia. k Data presented in part previously.^1^


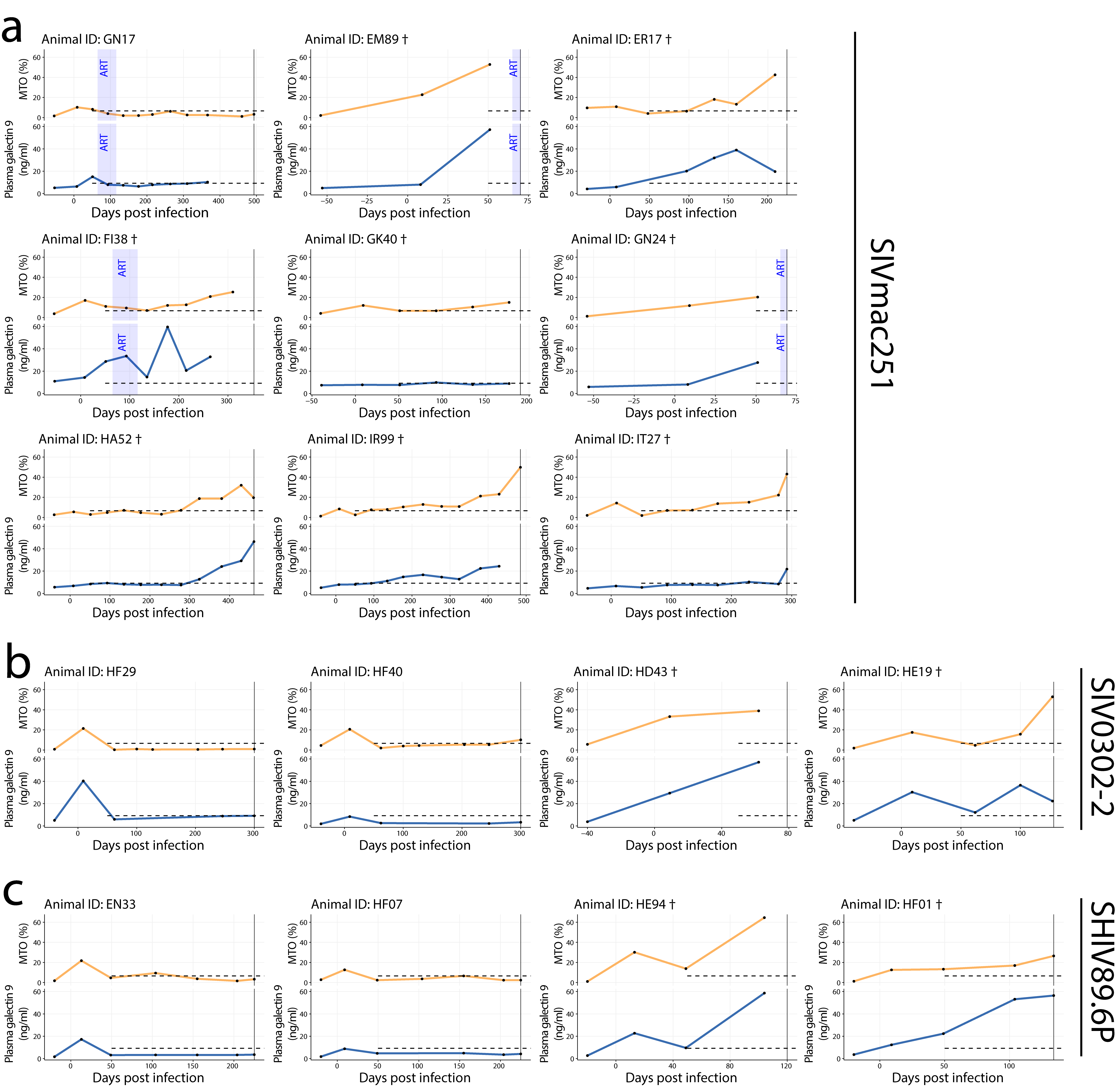


**Figure S2. MTO and plasma galectin-9 evolution throughout course of infection.** Longitudinal representation of MTO and galectin-9 as a function of days post infection for animals inoculated with SIVmac251 (a), SIV0302-2 (b), and SHIV89.6P (c). Thresholds calculated in Supplementary Fig. S4 predicting which animal would ultimately require euthanasia were displayed with a dashed line (6.6 % for MTO and 9.2 ng/ml for galectin-9). † = animal was euthanized. Solid lines represent the euthanasia date. The blue box represents the treatment with ART when provided.


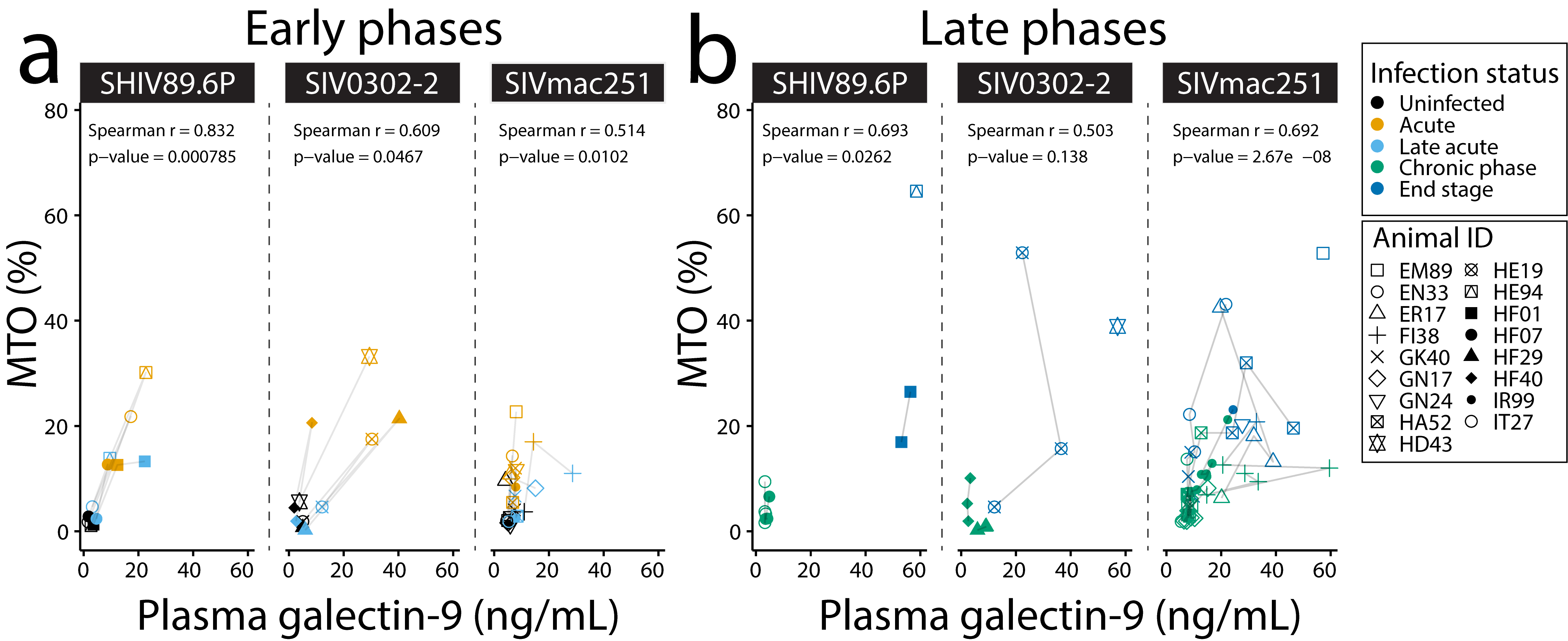


**Figure S3. Galectin-9 correlates with MTO in the early and late phases of SIV infection, regardless of the viral strain.** Correlation of Galectin-9 to MTO during early and late phase of SIV infection for animals infected with three different viral strains. Time points were classified based on the date of infection and cause of euthanasia (medical cull or end of study). Uninfected time points are from animals ranging from 54 to 40 days before infection. Acute phase data points were all measured at 9 DPI, except for EN33 and HE94, which were at 13 DPI. Late acute data represent the first time points after acute phase (ranging from 49 to 62 days post infection). Chronic phase time points were higher than 50 DPI and at least 100 days prior to euthanasia. End stage time points were less than 100 days prior to euthanasia. Data points between 50 and 63 DPI are present in both Panel a and b as Late acute and chronic stage, respectively.


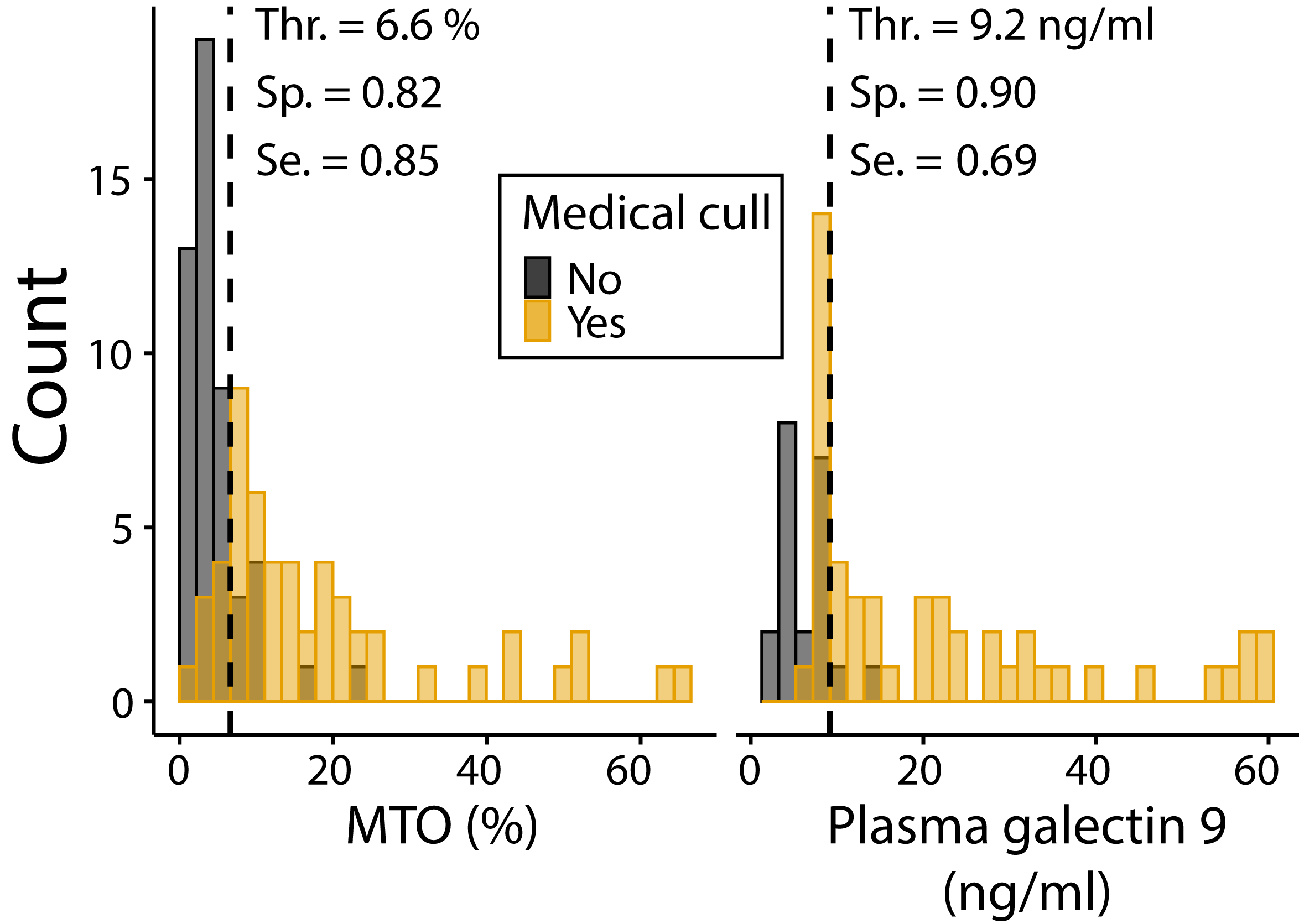


**Figure S4. Plasma galectin-9 appears relevant to predicting onset of disease progression.** Histogram of post-acute time points (DPI ≥ 50) of SIV-infected animals and colour-coded for the disease progression. Samples from animals ultimately progressing to euthanasia or not were regrouped and displayed in orange or grey histogram colours, respectively. The two best markers among the 10 tested to classify the samples were MTO (left) and plasma galectin-9 (right). ROC curves were calculated and the optimal thresholds were determined with the Youden's J statistic to maximize the sum of sensitivity and specificity. In case of tie, thresholds with the best specificity were selected. Dashed lines represent this threshold. Thr. = threshold value for measured parameter, Sp. = specificity at threshold, Se. = sensitivity at the threshold.


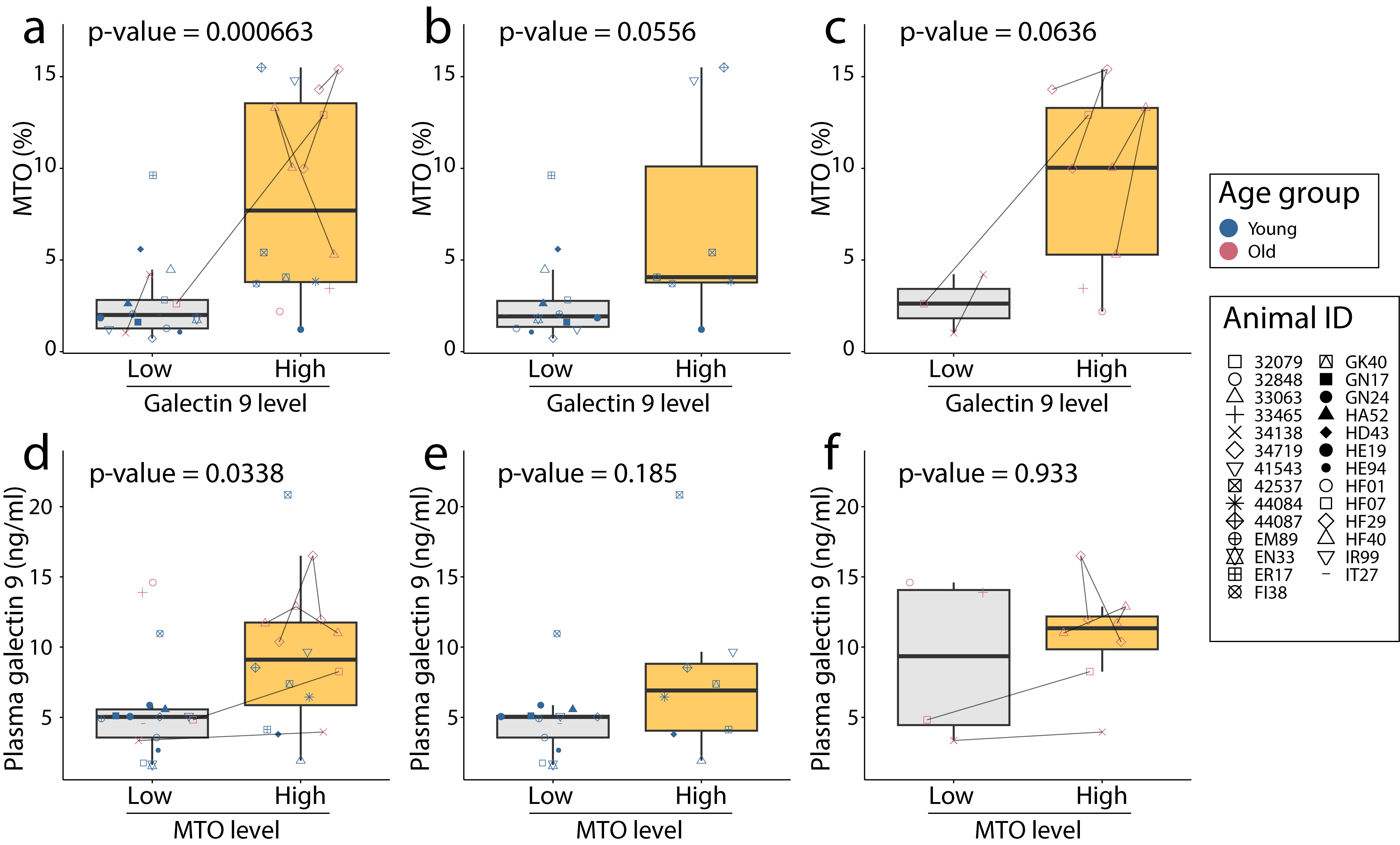


**Figure S5. Plasma galectin-9 and MTO levels shift together in uninfected animals.** Comparison of MTO or galectin-9 values between uninfected animal samples segregated in groups in relation to galectin-9 or MTO levels, respectively. (a-c) Comparison of MTO value between high or low galectin-9 groups. (d-f). Comparison of galectin-9 value between high or low MTO groups. (a&d) All age groups, (b&e) young animals (7-9 years old), (c&f) old animals (16-22 years old). Low and high groups correspond to lower and equal or higher than the median of all samples respectively. Median values were 3.71 % for MTO and 5.57 ng/ml for galectin-9. Values from the same animals were connected chronologically. Wilcoxon test p value is reported. The test was performed assuming independence between samples. Each box is comprised of the median, first and third quartile.

**Table S1.** Demographic information of the animals used in the study.

| **Animal ID** | **Sex** | **Age during study (years)** | **Age at infection (years)** | **Necropsy time (Days post infection)** | **Pathology findings** | **Viral strain** | **Euthanized** | **CD8 depletion** | **ART**  **Start-Stop (Days post infection)** | **Housing center** |
| --- | --- | --- | --- | --- | --- | --- | --- | --- | --- | --- |
| 32079 | F | 19 | / | / | / | / | / | No | / | CNPRC |
| 32848 | F | 18 | / | / | / | / | / | No | / | CNPRC |
| 33063 | M | 18 | / | / | / | / | / | No | / | CNPRC |
| 33465 | F | 18 | / | / | / | / | / | No | / | CNPRC |
| 34138 | F | 22 | / | / | / | / | / | No | / | CNPRC |
| 34719 | F | 16 | / | / | / | / | / | No | / | CNPRC |
| 41543 | M | 9 | / | / | / | / | / | No | / | CNPRC |
| 42537 | M | 8 | / | / | / | / | / | No | / | CNPRC |
| 44084 | M | 7 | / | / | / | / | / | No | / | CNPRC |
| 44087 | M | 7 | / | / | / | / | / | No | / | CNPRC |
| EM89 | M | 8 | 8.0 | 70 | nephrosis | SIVmac251 | Yes | Yes | 65-70 | TNPRC |
| EN33 | M | 9 | 9.3 | 225 | euthanasia | SHIV89.6P | No | No | / | TNPRC |
| ER17 | M | 8 | 7.7 | 224 | pneumonia | SIVmac251 | Yes | Yes | / | TNPRC |
| FI38 | M | 7 | 7.0 | 353 | dehydration and weight loss | SIVmac251 | Yes | Yes | 65-116 | TNPRC |
| GK40 | M | 5 | 5.0 | 190 | respiratory distress | SIVmac251 | Yes | No | / | TNPRC |
| GN17 | M | 5 | 5.0 | 493 | euthanasia | SIVmac251 | No | Yes | 65-116 | TNPRC |
| GN24 | M | 5 | 5.0 | 69 | nephrosis | SIVmac251 | Yes | Yes | 65-69 | TNPRC |
| HA52 | M | 4 | 4.2 | 461 | dehydration | SIVmac251 | Yes | No | / | TNPRC |
| HD43 | M | 5 | 5.2 | 79 | Gastro-enterocolitis | SIV0302-2 | Yes | No | / | TNPRC |
| HE19 | M | 5 | 5.2 | 128 | cytomegalovirus infection | SIV0302-2 | Yes | No | / | TNPRC |
| HE94 | M | 5 | 5.4 | 120 | euthanasia | SHIV89.6P | Yes | No | / | TNPRC |
| HF01 | M | 5 | 5.4 | 134 | pneumonia | SHIV89.6P | Yes | No | / | TNPRC |
| HF07 | M | 5 | 5.4 | 225 | euthanasia | SHIV89.6P | No | No | / | TNPRC |
| HF29 | M | 5 | 5.1 | 300 | Lympho-proliferative disease | SIV0302-2 | No | No | / | TNPRC |
| HF40 | M | 5 | 5.1 | 300 | euthanasia | SIV0302-2 | No | No | / | TNPRC |
| IR99 | M | 5 | 5.3 | 485 | encephalitis | SIVmac251 | Yes | No | / | TNPRC |
| IT27 | M | 5 | 4.9 | 292 | neoplasm lymphoma | SIVmac251 | Yes | No | / | TNPRC |

**Table S2. Antibodies used in the study.**

| **Antibody (conjugate; clone)** | **Source** | **Catalogue number** | **RRID** |
| --- | --- | --- | --- |
| Anti-CD3 (V500; SP34-2) | BD Biosciences | 560770 | AB_1937322 |
| Anti-CD3 (AF700; SP34-2) | BD Biosciences | 557917 | AB_396938 |
| Anti-CD3 (Pacific Blue; SP34-2) | BD Biosciences | 558124 | AB_397044 |
| Anti-CD3 (BV421; SP34-2) | BD Biosciences | 562877 | AB_2737860 |
| Anti-CD4 (APC-H7; L200) | BD Biosciences | 560837 | AB_10563933 |
| Anti-CD8 (V500; SK1) | BD Biosciences | 561618 | AB_10895808 |
| Anti-CD8 (BV650; SK1) | BD Biosciences | 565289 | AB_2739158 |
| Anti-CD8α (Cell depleting) | NHP Nonhuman Primate Reagent Resources | MT807R1 | AB_2716320 |
| Anti-CD1c (APC; AD5-8E7) | Militenyi | 130-113-299 | AB_2726078 |
| Anti-CD11c (AF700; 3.9) | eBioscience | 56-0116-42 | AB_10547281 |
| Anti-CD14 (Pacific Blue; M5E2) | BD Biosciences | 558121 | AB_397041 |
| Anti-CD14 (BV711; M5E2) | BD Biosciences | 740773 | AB_2740436 |
| Anti-CD14 (ECD; RMO52) | Beckman Coulter | IM2707U | AB_130853 |
| Anti-CD16 (APC-H7; 3G8) | BD Biosciences | 560715 | AB_1727432 |
| Anti-CD16 (BV510; 3G8) | BD Biosciences | 563829 | AB_2744296 |
| Anti-CD20 (eFluor 450; 2H7) | ThermoFisher, eBioscience | 48-0209-42 | AB_1633384 |
| Anti-CD20 (PE-CF594; 2H7) | BD Bioscience | 562550 | AB_2737646 |
| Anti-CD20 (APC-H7; L27) | BD Bioscience | 641396 | AB_1645724 |
| Anti-CD20 (ECD; B9E9) | Beckman Coulter | B92433 | AB_2625660 |
| Anti-CD28 (PE-CF594; 28.2) | BD Biosciences | 562323 | AB_11153681 |
| Anti-CD28 (ECD; 28.2) | Beckman Coulter | 6607111 | AB_1575955 |
| Anti-CD45 (APC; MB4-6D6) | Miltenyi | 130-123-787 | AB_2819522 |
| Anti-CD95 (APC; DX2) | BD Biosciences | 558814 | AB_398659 |
| Anti-CD123 (PCP-Cy5.5; 7G3) | BD Biosciences | 560087 | AB_1645454 |
| Anti-CD163 (PE; Mac 2-158) | Trillium | IQP-570R | AB_2933960 |
| Anti-HLA-DR (PE-Cy7; L243) | BD Biosciences | 335795 | AB_399973 |
| Anti-BrdU (FITC; 3D4) | BD Biosciences | 556028 | AB_396304 |
| Anti-BrdU (PCP-Cy5.5; 3D4) | BD Biosciences | 560809 | AB_2033929 |
| Anti-CCR5 (PE; 3A9) | BD Biosciences | 550632 | AB_2072548 |
| Anti-CCR7 (V450; 150503) | BD Biosciences | 560863 | AB_2033952 |

**Table S3. List of markers evaluated in the study.**

| **CBC** | **Chem value** | **Flow cytometry^1^** | | **Miscellaneous** |
| --- | --- | --- | --- | --- |
|  |  | **Cell type** | **Marker used** |  |
| White Blood Cell | Sodium | CD4+ T-cells | SSC-A low, HLADR-, CD3+, CD4+ | Plasma viral load (RT-qPCR) |
| Red Blood Cell | Potassium | CM CD4 T-cells | SSC-A low, HLADR-, CD95+, CD28+, CD3+, CD4+ | Weight |
| Haemoglobin | Chloride | CD8+ T-cells | SSC-A low, HLADR-, CD3+, CD8+ | Plasma Galectin-9 (ELISA) |
| Haematocrit | Total protein | CD4+ CD8+ T-cells | SSC-A low, HLADR-, CD3+, CD4+, CD8+ | Plasma Galectin-3 (ELISA) |
| Mean Corpuscular Volume | Albumin |  |  | Plasma sCD163 (ELISA) |
| Mean Corpuscular Haemoglobin | Globulin | NK-cells | SSC-A low, HLADR-, CD3-, CD8+ | Days post infection |
| Mean Corpuscular Haemoglobin Concentration | Albumin/Globulin |  |  | Days to euthanasia |
| Red Cell Distribution Width | Calcium | B-cells | CD3-, HLA-DR+, CD20+ |  |
| Platelets | Bilirubin (total) | Granulocytes | SSC-A high |  |
| Mean platelet volume | Blood urea nitrogen | Basophils | SSC-A high, CD123+ |  |
| reticulocyte | Glucose | Eosinophils | SSC-A high, auto fluorescence high |  |
| % Segmented Neutrophils | Creatinine | Neutrophils | SSC-A high, auto fluorescence low, CD123- |  |
| % Lymphocytes | Phosphorous |  |  |  |
| % Monocytes | Alkaline phosphatase | CD1c DC | SSC-A int, HLADR+, CD20-, CD14-, CD16-, CD11c-, CD163-, CD123-CD1c+ |  |
| % Eosinophils | Aspartate transaminase | CD123 DC | SSC-A int, HLADR+, CD20-, CD14-, CD16-, CD11c-, CD163-, CD123+, CD1c- |  |
| % Basophils | Alanine aminotransferase | Classical monocytes | SSC-A int, HLADR+, CD20-, CD14+, CD16- |  |
| Segmented Neutrophils count | Lactate dehydrogenase | Intermediate monocytes | SSC-A int, HLADR+, CD20-, CD14+, CD16+ |  |
| Lymphocytes count | Creatine kinase | non-classical monocytes | SSC-A int, HLADR+, CD20-, CD14 low, CD16- |  |
| Monocytes count | Cholesterol |  |  |  |
| Eosinophils count | CO_2_ |  |  |  |
| Basophils count | Blood urea nitrogen/Creatine |  |  |  |
|  | Triglyceride |  |  |  |
|  | Gamma-glutamyl transferase |  |  |  |
| 1. CD163 and CD11c presence were monitored in monocytes populations  CCR7 and CCR5 presence were monitored in T cell populations  BrdU was monitored in all populations (all samples are one day post pulse)  MTO was calculated as the percent of CD14+ monocytes (classical and intermediate) positive for BrdU one day post pulse  CD14 and CD16 MFI were calculated for all populations  Some values were normalized on other population (MFI, or ratio of population) | | | | |

| **Table S4. Markers of MTO levels** | | | | |
| --- | --- | --- | --- | --- |
| **Marker** | **AUC** | **Threshold** | **Threshold specificity** | **Threshold sensitivity** |
| Plasma galectin-9 (ng/ml) | **0.86** | 18.2 | 0.88 | 0.77 |
| Plasma viral load (Log10(copies/ml)) | **0.79** | 6.7 | 0.75 | 0.75 |
| % CD4 CM among T−cells | 0.76 | 6.5 | 0.81 | 0.71 |
| % Lymphocytes (from CBC) | 0.75 | 33.6 | 0.75 | 0.64 |
| % Granulocytes (from flow cytometry) | 0.74 | 52.7 | 0.65 | 0.73 |
| % Neutrophils (from CBC) | 0.74 | 49.6 | 0.61 | 0.79 |
| Ratio CD4+/CD8+ T−cells | 0.63 | 0.6 | 0.55 | 0.78 |
| Days post infection | 0.62 | 255.0 | 0.87 | 0.39 |
| CD14 MFI of n−c monocytes  (normalized on T cells CD14 MFI) | 0.61 | 1.7 | 0.40 | 0.90 |

| **Table S5. Markers of nearing euthanasia** | | | | |
| --- | --- | --- | --- | --- |
| **Marker** | **AUC** | **Threshold** | **Threshold specificity** | **Threshold sensitivity** |
| MTO | **0.94** | 13.05 | 0.90 | 0.91 |
| Plasma galectin-9 (ng/ml) | **0.81** | 18.16 | 0.78 | 0.74 |
| % CD4 CM among T−cells | 0.78 | 52.65 | 0.70 | 0.81 |
| % Lymphocytes (from CBC) | 0.76 | 5.66 | 0.78 | 0.67 |
| % Neutrophils (from CBC) | 0.75 | 32.05 | 0.75 | 0.73 |
| % Granulocytes (from flow cytometry) | 0.75 | 55.05 | 0.76 | 0.65 |
| Plasma viral load [Log10(copies/ml)] | 0.72 | 1.71 | 0.48 | 0.93 |
| CD14 MFI of n−c monocytes  (normalized on T cells CD14 MFI) | 0.68 | 6.53 | 0.62 | 0.71 |
| Days post infection | 0.58 | 384.50 | 0.99 | 0.19 |
| Ratio CD4+/CD8+ T−cells | 0.57 | 0.35 | 0.82 | 0.56 |

| **Table S6. Markers of future disease progression** | | | | |
| --- | --- | --- | --- | --- |
| **Marker** | **AUC** | **Threshold** | **Threshold specificity** | **Threshold sensitivity** |
| MTO | **0.89** | 6.6 | 0.82 | 0.85 |
| Plasma galectin-9 (ng/ml) | **0.89** | 9.2 | 0.90 | 0.69 |
| % CD4 CM among T−cells | 0.81 | 8.3 | 0.85 | 0.69 |
| Plasma viral load (Log10(copies/ml)) | 0.72 | 5.1 | 0.36 | 0.95 |
| CD14 MFI of n−c monocytes  (normalized on T cells CD14 MFI) | 0.68 | 2.1 | 0.54 | 0.82 |
| % Lymphocytes (from CBC) | 0.65 | 36.2 | 0.63 | 0.61 |
| % Neutrophils (from CBC) | 0.60 | 40.3 | 0.32 | 0.88 |
| Ratio CD4+/CD8+ T−cells | 0.56 | 0.6 | 0.61 | 0.68 |
| Days post infection | 0.50 | 144.5 | 0.60 | 0.48 |
| % Granulocytes (from flow cytometry) | 0.48 | 54.0 | 0.43 | 0.67 |

# References

1 Takahashi N, Ardeshir A, Holder GE, Cai Y, Sugimoto C, Mori K *et al.* Comparison of predictors for terminal disease progression in simian immunodeficiency virus/simian-HIV-infected rhesus macaques. *AIDS* 2021; **35**: 1021–1029.
